# Stochastic modeling of obesity status in United States adults using Markov Chains: A nationally representative analysis of population health data from 2017–2020

**DOI:** 10.1002/osp4.697

**Published:** 2023-07-27

**Authors:** Alexander A. Huang, Samuel Y. Huang

**Affiliations:** ^1^ Cornell University Ithaca New York USA; ^2^ Northwestern University Feinberg School of Medicine Chicago Illinois USA; ^3^ Virginia Commonwealth University School of Medicine Richmond Virginia USA

**Keywords:** Markov chain, NHANES, obesity

## Abstract

**Importance:**

The prevalence of obesity among United States adults has increased from 34.9% in 2013–2014 to 42.8% in 2017–2018. Developing methods to model the increase of obesity over‐time is a necessity to know how to accurately quantify its cost and to develop solutions to combat this national public health emergency.

**Methods:**

A cross‐sectional cohort study using the publicly available National Health and Nutrition Examination Survey (NHANES 2017–2020) was conducted in individuals who completed the weight questionnaire and had accurate data for both weight at the time of survey and weight 10 years ago. To model the dynamics of obesity, a Markov transition state matrix was created, which allowed for the analysis of weight transitions over time. Bootstrap simulation was incorporated to account for uncertainty and generate multiple simulated datasets, providing a more robust estimation of the prevalence and trends in obesity within the cohort.

**Results:**

Of the 6146 individuals who met the inclusion criteria, 3024 (49%) individuals were male and 3122 (51%) were female. There were 2252 (37%) White individuals, 1257 (20%) Hispanic individuals, 1636 (37%) Black individuals, and 739 (12%) Asian individuals. The average BMI was 30.16 (SD = 7.15), the average weight was 83.67 kilos (SD = 22.04), and the average weight change was a 3.27 kg (SD = 14.97) increase in body weight. A total of 2411 (39%) individuals lost weight, and 3735 (61%) individuals gained weight. 87 (1%) individuals were underweight (BMI <18.5), 2058 (33%) were normal weight (18.5 ≤ BMI <25), 1376 (22%) were overweight (25 ≤ BMI <30) and 2625 (43%) were in the obese category (BMI >30).

**Conclusion:**

United States adults are at risk of transitioning from normal weight to the overweight or obese category. Markov modeling combined with bootstrap simulations can accurately model long‐term weight status.

## Graphical abstract

The article reports on a retrospective cross‐sectional cohort study using the National Health and Nutrition Examination Survey to model the increase of obesity over time. The study found that 61% of patients gained weight, and United States adults are at risk of transitioning from normal weight to becoming overweight or obese. The authors suggest that Markov modeling combined with bootstrap simulations can accurately model long‐term weight status.

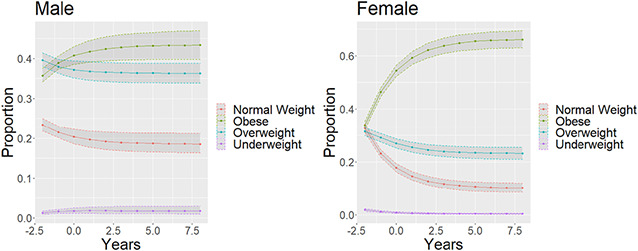

## 1 INTRODUCTION

The prevalence of obesity in the United States has reached alarming levels, posing a significant global health concern.^1^ Recent data from the National Health and Nutrition Examination Survey (NHANES) reveals a substantial increase in obesity rates between 2013 and 2018. The prevalence of adult obesity rose from 34.9% in 2013‐2014 to 42.8% in 2017–2018, representing a significant jump.^2^ These statistics paint a grim picture of a rapidly expanding disease with far‐reaching consequences.

To address the urgent national public health issue of obesity and accurately estimate its cost, it is crucial to model the trajectory of obesity over time.^3^, ^4^, ^5^ However, there is a lack of studies that specifically investigate long‐term weight gain and develop projection models, despite its recognized significance as a public health concern. Therefore, this study aimed to provide a comprehensive analysis of long‐term weight fluctuations in adult individuals in the United States using Markov chains. In addition, innovative simulation techniques will be introduced to accurately predict the impact of obesity on the future adult population of the United States. Markov chains, combined with bootstrap simulation, are valuable tools for modeling obesity. Markov chains are mathematical models that describe a system's transition from one state to another based on probabilities. In the context of obesity, the mentioned states represent distinct weight categories, including the underweight, normal weight, overweight, and the obese category. Analyzing the transitions between these weight categories over time using Markov chains provides valuable insights into the probabilities of individuals moving between categories. This approach helps to comprehend the dynamics of obesity and make projections about future weight distributions.

Bootstrap simulation, on the other hand, is a resampling technique that involves generating multiple simulated datasets by repeatedly sampling from the original dataset.^6^, ^7^ Through the application of bootstrap simulation, multiple transition matrices can be generated based on the Markov chain's transition matrix, thereby capturing the uncertainty in the estimates.^8^ These simulated transition matrices allow for the projection of future weight category distributions and estimation of the proportion of individuals in each category. By incorporating uncertainty through bootstrap simulation, confidence intervals can be obtained, providing a measure of the reliability of the results. The combination of Markov chains and bootstrap simulation presents a robust methodology for modeling obesity, as it considers the probabilistic nature of weight transitions and accounts for data uncertainty.^9^ This approach enhances the general understanding of the obesity epidemic and can inform evidence‐based interventions and policies aimed at addressing this significant public health issue.

This study aimed to fill the knowledge gap in obesity epidemiology by utilizing a comprehensive multistage cross‐sectional dataset representative of the US population, employing Markov Chains and bootstrap simulation techniques to provide valuable estimates of obesity rates and insights into the population's transition between different stages.

## 2 METHODS

A cross‐sectional cohort study using the publicly available National Health and Nutrition Examination Survey (NHANES 2017–2018 and 2019–2020)^10^, ^11^, ^12^, ^13^ was conducted in individuals who completed the weight questionnaire and had accurate data for both weight at the time of survey and weight 10 years ago.

### 2.1 Ethics approval and consent to participate

The acquisition and analysis of the data within this study was approved by the National Center for Health Statistics Ethics Review Board.

### 2.2 Dataset and cohort selection

The National Health and Nutrition Examination Survey (NHANES) is a program designed by the National Center for Health Statistics (NCHS), which has been leveraged to assess the health and nutritional status of the United States population. The NHANES dataset is a series of cross‐sectional, complex, multi‐stage surveys conducted by the Centers for Disease Control and Prevention (CDC) on a nationally representative cohort of the United States population to provide health, nutritional, and physical activity data. In the present study, adult (≥18 years old) individuals in the NHANES dataset were included if they completed the weight questionnaire, leading to the inclusion of 6146 total individuals.

### 2.3 Assessment of long‐term weight change

From the questionnaire dataset in NHANES, the weight 10 years prior to the study and the current weight of the patient were extracted. The difference between the patient's weight and the weight 10 years ago was the metric used for Long‐term Weight Change within this study. Weight categories defined by the Centers of Disease Control and Prevention (CDC) were utilized in this study: Individuals who had a BMI less than 18.5 were considered underweight, individuals with BMI between 18.5 and 25 were considered of normal weight, individuals with BMI between 25 and 30 were overweight, and individuals with BMI above 30 were considered in the obese category.

### 2.4 Model construction and statistical analysis

Demographic variables were calculated for all individuals, those that lost weight, and those that gained weight. The *p*‐values were calculated using two‐sided *t*‐tests for continuous variables and chi‐square tests for categorical variables to compare the differences between the two groups: individuals who lost weight and those who gained or maintained weight. Dumbbell plots, stratified by race and gender, were utilized to compare the weight of the patient compared to their weight 10 years ago. Paired *t*‐tests were used to compare whether there was a change in weight over the 10‐year period. Additionally, individuals were classified as underweight, normal weight, overweight, or obese at both the time of survey and 10 years prior. To account for the complex survey design and ensure population representativeness, sampling weights provided by NHANES were utilized in the estimation of the initial and final transition states. The weights were incorporated to adjust for the probability of selection and nonresponse bias. Specifically, the weight variables were applied when calculating the proportions or probabilities of the initial and final states, providing accurate estimates at the population level. This allowed for a more robust analysis of the Markov chain transition states, considering the weight‐adjusted distribution of individuals in the population. A transition matrix was built based upon this cohort and Markov Chains were used to project which states (underweight, normal weight, overweight, or obese) the patient was likely to be in for the next 10 decades. These projections were plotted visually in line‐graphs. Confidence intervals for these estimates were obtained through bootstrap simulation of the transition matrices. All data analyses were conducted using R version 4.2.2 (2022‐10‐31) (R Foundation Vienna, Austria) with NHANES‐provided sampling weights.

## 3 RESULTS

**FIGURE 1:**
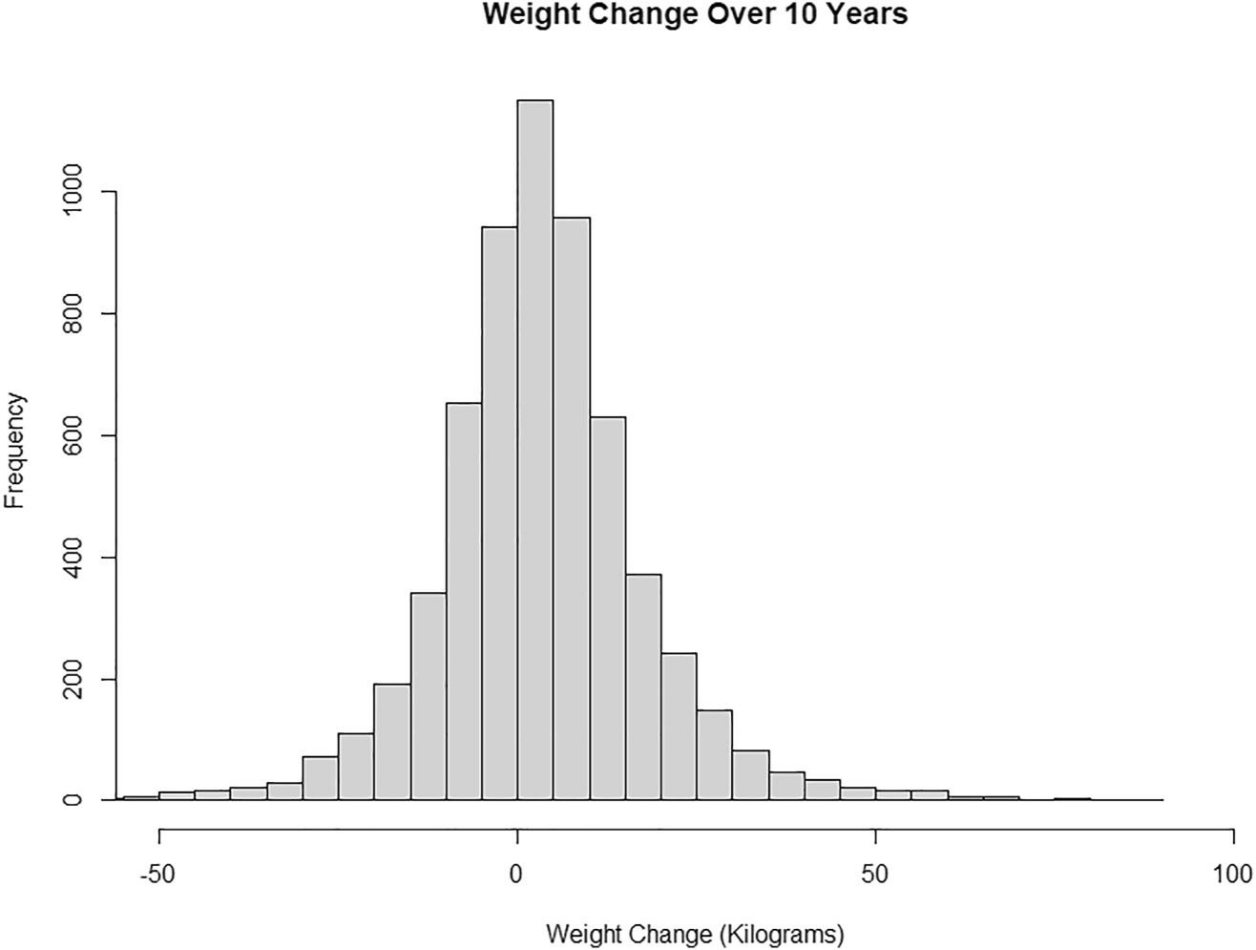
Histogram of 10‐year weight change (in kilograms).

Of the 6146 individuals who met the inclusion criteria, 3024 (49%) individuals were male and 3122 (51%) were female. There were 2252 (37%) White individuals, 1257 (20%) Hispanic individuals, 1636 (37%) Black individuals, and 739 (12%) Asian individuals. The average BMI was 30.16 (SD = 7.15), the average weight was 83.67 kilos (SD = 22.04), and the average weight change was a 3.27 kg (SD = 14.97) increase in body weight (Figure 1). A total of 2411 (39%) individuals lost weight, and 3735 (61%) individuals gained weight (Table 1). 87 (1%) individuals were underweight (BMI <18.5), 2058 (33%) were normal weight (18.5 ≤ BMI <25), 1376 (22%) were overweight (25 ≤ BMI <30) and 2625 (43%) were obese (BMI >30).

**TABLE 1:** Summary of patient characteristics (demographic, socioeconomic, and laboratory measurements), stratified by individuals with weight loss versus no weight loss.

	All patients	Lost weight	Gained or maintained weigh	*p*‐value
Total patients (*N*)	6146 (1)	2411 (0.39)	3735 (0.61)	N/A
Age; mean (SD)	58.39 (12.94)	62.01 (12.85)	56.05 (12.45)	*p* < 0.001
Gender male; count (%)	3024 (0.49)	1376 (0.57)	1648 (0.44)	*p* < 0.001
Gender female; count (%)	3122 (0.51)	1035 (0.43)	2087 (0.56)	
Race white; count (%)	2252 (0.37)	943 (0.39)	1309 (0.35)	*p* < 0.001
Race other; count (%)	262 (0.04)	113 (0.05)	149 (0.04)	
Race hispanic; count (%)	1257 (0.2)	462 (0.19)	795 (0.21)	
Race black; count (%)	1636 (0.27)	609 (0.25)	1027 (0.27)	
Race Asian; count (%)	739 (0.12)	284 (0.12)	455 (0.12)	
Income_Poverty_Ratio; mean (SD)	2.7 (1.63)	2.59 (1.61)	2.77 (1.64)	*p* = 0.53
Weight (kg); mean (SD)	83.67 (22.04)	76.89 (18.94)	88.04 (22.79)	*p* < 0.001
Weight change; mean (SD)	3.27 (14.97)	−9.71 (10.39)	11.64 (10.96)	*p* < 0.001
Body Mass index (kg/m^2); mean (SD)	30.16 (7.15)	27.58 (5.96)	31.82 (7.36)	*p* < 0.001
Direct HDL cholesterol (mg/dL); mean (SD)	53.99 (16.34)	56.22 (17.36)	52.56 (15.48)	*p* < 0.001
LDL cholesterol, friedewald (mg/dL); mean (SD)	110.89 (37.05)	105.63 (36.74)	114.41 (36.84)	*p* < 0.001
Cholesterol, refrigerated serum (mg/dL); mean (SD)	189.64 (41.88)	184.65 (43.57)	192.86 (40.44)	*p* < 0.001
Triglyceride (mg/dL); mean (SD)	114.94 (97.1)	105.21 (76.42)	121.43 (108.26)	*p* < 0.001
Albumin, urine (mg/L); mean (SD)	60.16 (367.89)	82.35 (490.35)	45.95 (259.97)	*p* < 0.001
Creatinine, urine (mg/dL); mean (SD)	121.99 (80.62)	116.61 (77.26)	125.44 (82.52)	*p* < 0.001
Albumin creatinine ratio (mg/g); mean (SD)	61.17 (396.2)	83.55 (517.76)	46.85 (292.24)	*p* < 0.001
Total cholesterol (mg/dL); mean (SD)	189.33 (41.85)	184.25 (43.52)	192.6 (40.41)	*p* < 0.001
Platelet count (1000 cells/uL); mean (SD)	241.76 (65.7)	234.72 (67.21)	246.32 (64.3)	*p* < 0.001
Insulin (pmol/L); mean (SD)	90.79 (150.55)	75.21 (129.96)	101.13 (161.99)	*p* < 0.001
Iron frozen, serum (ug/dL); mean (SD)	85.5 (34.49)	86.85 (35.02)	84.62 (34.12)	*p* < 0.001
Fasting glucose (mg/dL); mean (SD)	117.65 (40.76)	121.14 (46.42)	115.32 (36.34)	*p* < 0.001
Albumin, refrigerated serum (g/dL); mean (SD)	4.03 (0.33)	4.04 (0.34)	4.02 (0.32)	*p* < 0.001
Glucose, refrigerated serum (mg/dL); mean (SD)	106.12 (40.39)	110 (48.29)	103.61 (34.14)	*p* < 0.001
Phosphorus (mg/dL); mean (SD)	3.55 (0.53)	3.58 (0.55)	3.52 (0.51)	*p* < 0.001
Uric acid (mg/dL); mean (SD)	5.48 (1.48)	5.39 (1.49)	5.54 (1.48)	*p* < 0.001
Used any tobacco product last 5 days?; mean (SD)	1249 (0.2)	565 (0.23)	684 (0.18)	*p* < 0.001

**FIGURE 2:**
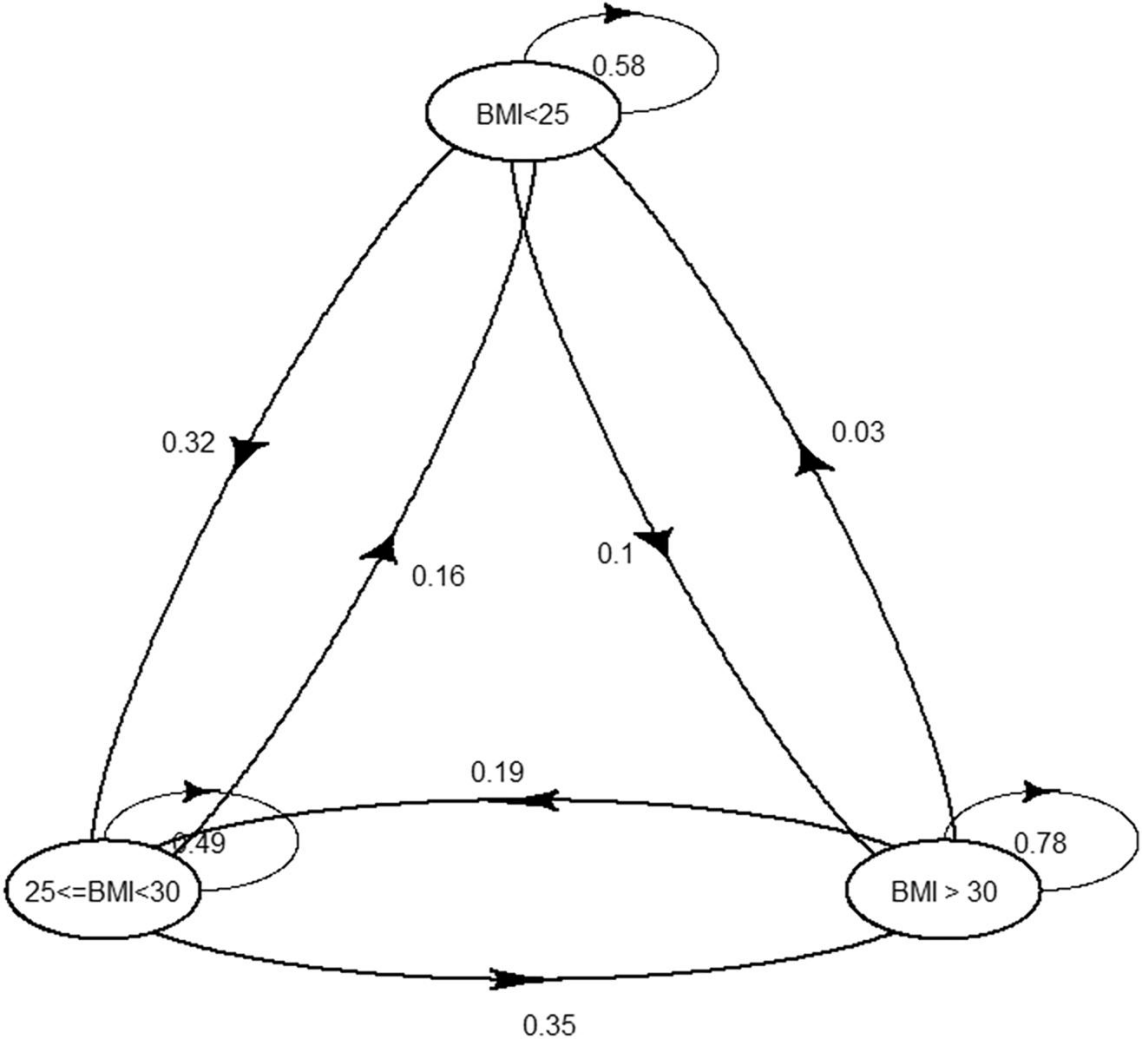
Transitions between Obese, Overweight, and Normal/Underweight. Each circle represents a specific BMI category, and arrows represent the proportion of one group transitioning to another group over the 10‐year period.

From the analysis of the transitions between normal/underweight, overweight, and obese, after 10 years, individuals who were normal/underweight stayed normal/underweight 58% of the time, became overweight 32% of the time, and became obese 10% of the time. Individuals who were overweight stayed overweight 49% of the time, became underweight 16% of the time, and became obese 35% of the time. Individuals who were obese stayed obese 78% of the time, became normal/underweight 3% of the time, and became overweight 19% of the time (Figure 2).

From projections overtime, individuals are more likely to end up in the obese weight category overtime. Additionally, there were significant sex‐differences (*p* < 0.001), with female individuals having a significantly higher proportion of individuals in the obese group in the long‐term projection. Upon visual inspection of Figure 5, compared to White individuals, Black individuals and Hispanic individuals had increased rates of obesity, while Asian individuals had a decreased rate of obesity in the long‐term projections. Furthermore, Asian individuals were the only individuals who showed a decrease in the obesity rate overtime in the long‐term projection graphs.

## 4 DISCUSSION

In this retrospective cross sectional cohort of United States adults, individuals on average gained 3.27 kg over the 10 year period. As a result of this age‐related weight gain, a large number of individuals who were normal weight will transition to become overweight or obese within a decade. Females were more likely to gain weight than males, Black and Hispanic individuals were more likely to gain weight than White individuals, and Asian individuals were less likely to gain weight than White individuals. These sex and race differences in weight change are concordant with previous epidemiological studies that studied sex‐ and race‐based differences in gradual weight gain. These overall trends in weight gain match other analyses of both NHANES data and other cohorts of the United States population.^14^, ^15^, ^16^, ^17^, ^18^, ^19^, ^20^, ^21^, ^22^

**FIGURE 3:**
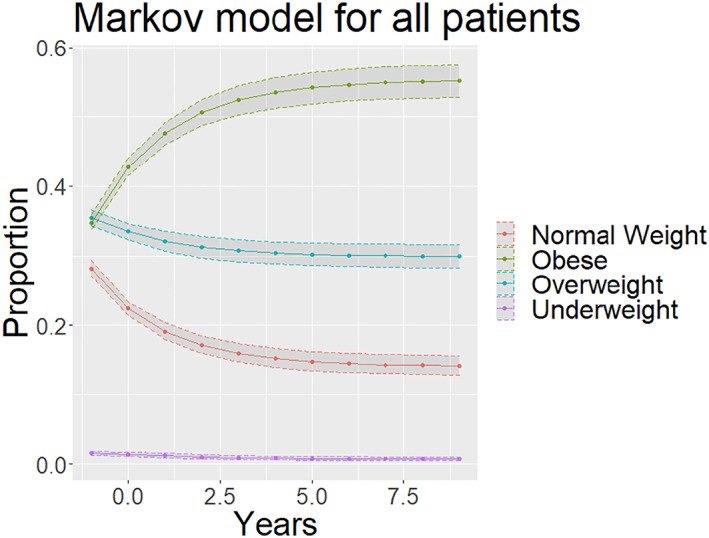
Markov model of weight overtime—each new dot represents a decade. *X*‐axis years in units of 10 years. *Y*‐axis is the prevalence of each weight category.

**FIGURE 4:**
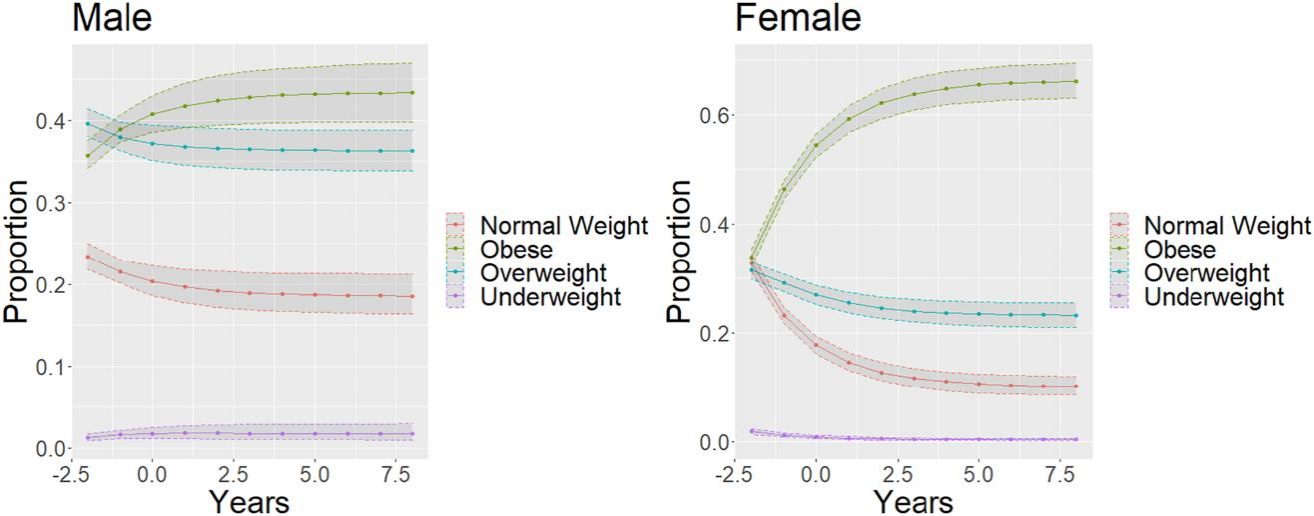
Sex Differences in Weight overtime—each new dot represents a decade. *X*‐axis years in units of 10 years. *Y*‐axis is the prevalence of each weight category.

**FIGURE 5:**
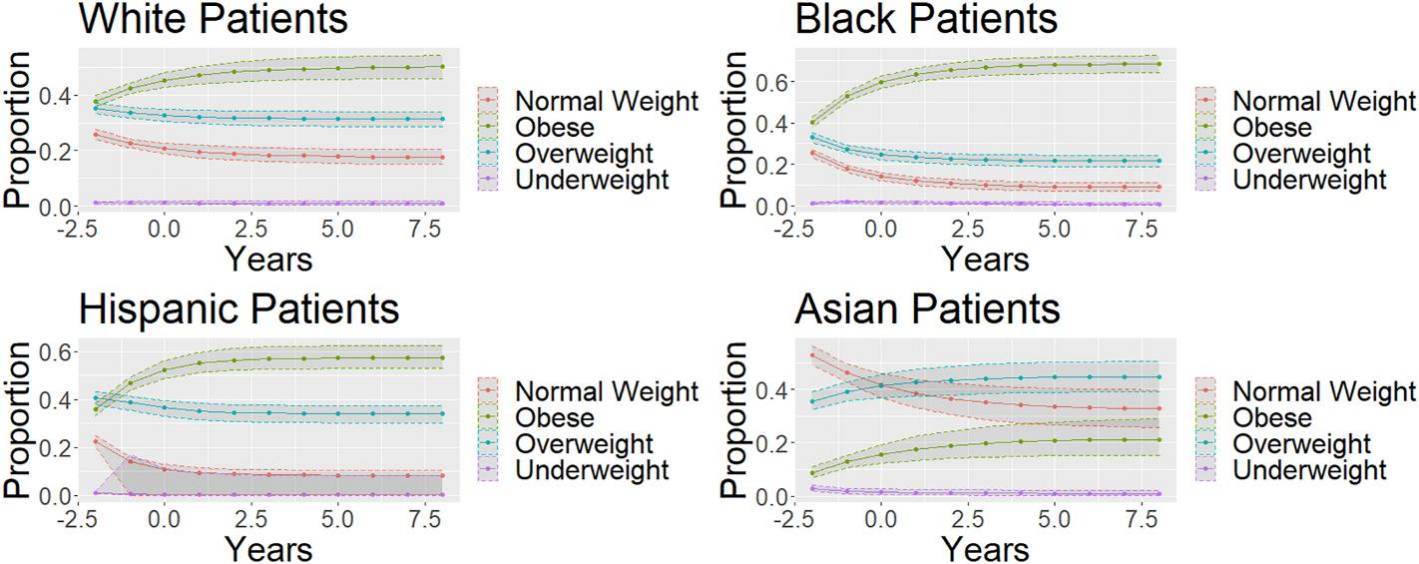
Race Differences in Weight overtime—each new dot represents a decade. *X*‐axis years in units of 10 years. *Y*‐axis is the prevalence of each weight category.

Many past obesity studies have observed age‐related weight gain in population cohorts.^23^, ^24^, ^25^, ^26^ These studies showed significant weight gain in the populations (greater than 3 kg per decade) and were found to have significant racial and sex‐based disparities.^27^, ^28^, ^29^, ^30^ What the present study contributes to the literature is a novel method for modeling obesity trends over time. Markov chains were utilized to observe the trajectories of the cohort.^31^, ^32^, ^33^ The models allowed precise projections into the future (Figure 3).^34^, ^35^, ^36^ Additionally, the novel use of bootstrap simulations allowed for accurate quantification of the variance within these Markov chain projections (Figures 3, 4, 5).^37^, ^38^, ^39^, ^40^ Other studies that have attempted to quantify the variability present in Markov chain predictions focus on the transition matrix, with each transition probability having a confidence interval.^41^ However, since the matrix generated 10 subsequent predictions, small initial errors would be compounded.^42^, ^43^, ^44^, ^45^ For example, a 5% error would then be compounded to a 62.8% error after 10 iterations. The analysis utilized bootstrap simulation to generate 10,000 unique Markov chains from the data and generated accurate confidence intervals from these 10,000 generations, allowing us to have confidence of accurately estimating the true variance and considering the compounding error that is present from each subsequent projection into the future.

Subgroup analysis allowed further comparisons of long‐term obesity states for different subpopulations (Figures 3 and 5). Through the Markov chains, it was observed that Black and Hispanic individuals were more likely to be in the obese category in the long term compared to White individuals, and Asian individuals were less likely to be in the obese category compared to White individuals (Figure 5). Furthermore, Female individuals were more likely to transition to the obese category than Male individuals (Figure 4). These findings were concordant with other epidemiological studies, which serve to validate this approach in long‐term projections into the future.

Lastly, one powerful statistic of Markov chains is the stationary distribution: the long‐term state of the chain. This can be visualized within figures, where it was observed that the distribution does not seem to change from decade to decade. This represents the long‐term distribution. The Markov chain within this study will thus demonstrate that without significant interventions to change the trajectory of gradual weight gain in United States adults, the majority of individuals will end up overweight and obese and suffer from preventative early mortality and significant morbidities linked with diabetes and heart disease.

This study has several strengths and weaknesses. The utilization of the National Health and Nutrition Examination Survey (NHANES) dataset represents a significant strength of this study. NHANES is a nationally representative cross‐sectional study that employs a robust sampling methodology. The survey enrolls participants through stratified multistage probability and oversampling design, ensuring that the dataset accurately reflects the characteristics of the civilian noninstitutionalized population in the United States. This approach allows for weighted analysis, enhancing the generalizability and representativeness of the study findings. By utilizing the NHANES data, along with the application of Markov Chains and bootstrap simulation, this study can provide reliable and accurate estimates of trends in adult obesity, as well as valuable insights into the prevalence of overweight and obesity among various demographic groups.

As a result, the study carries the limitations of cross‐sectional studies. However, this study allows for the selection of a large cohort, evaluation of data quality, and due to the publicly available nature of the cohort, allows for increased replication and follow‐up studies based upon the same cohort. Furthermore, the cohort relied on surveys to obtain the outcome of interest (weight 10 years ago) as well as the dietary and lifestyle information. More accurate measurements may have been achieved with prospective studies with lab measurements of weight, but these may interfere with natural patient habits since they know they are part of a nutrition study and may have significantly different behaviors than the general population. Additionally, self‐reported survey information allows for an increased volume of participants to be included within this study. Another weakness was the voluntary nature of this cohort, with participants choosing to opt into the study instead of being randomly selected. This may artificially select a different cohort that may significantly differ from the population. However, the analysis found a demographically diverse population, so these results may still be generalizable to other cohorts.

## 5 CONCLUSION

United States adults are at risk of transitioning from normal weight to becoming overweight or obese category. Markov modeling combined with bootstrap simulations can accurately model long‐term weight status.

## AUTHOR CONTRIBUTIONS

Alexander Huang took part in study design, formal analysis, and writing. Samuel Huang took part in study design and writing.

## CONFLICT OF INTEREST STATEMENT

Authors have no competing interests.

## CONSENT FOR PUBLICATION

The authors provide consent to publish this work.
